# Pediatric axillary artery aneurysm

**DOI:** 10.1016/j.jvscit.2025.101754

**Published:** 2025-02-19

**Authors:** Thomas Boland, Bernadette Aulivola

**Affiliations:** a Stritch School of Medicine, Loyola University Chicago, Maywood, IL; b Division of Vascular Surgery and Endovascular Therapy, Department of Surgery, Loyola University Medical Center, Maywood, IL

**Keywords:** Pediatric, Aneurysm, Axillary artery

## Abstract

Nonaortic aneurysms are rare in the pediatric patient population with axillary artery aneurysms accounting for <5% of these. The most common cause of pediatric nonaortic aneurysm is trauma followed by arterial dysplasia. There are only 14 reported cases of idiopathic true axillary aneurysm in the pediatric patient. We present a rare case of an idiopathic isolated right axillary artery aneurysm in a 13-year-old girl, managed with surgical repair with autogenous venous interposition. A thorough workup should be performed in pediatric patients with peripheral aneurysms to determine the etiology, which may guide the clinical management.

Nonaortic aneurysms are rare in the pediatric population with axillary artery aneurysms accounting for a small fraction based on published data. Childhood aneurysmal disease etiologies include infection, giant cell aortoarteritis, autoimmune connective tissue disease, Kawasaki disease, Ehlers-Danlos syndrome, Marfan's syndrome, noninflammatory medial degeneration, arterial dysplasia, congenital idiopathic factors, and vascular injury.^1^ Idiopathic aneurysms are especially rare, with only about 14 reports of axillary artery aneurysms in the English language literature to date.^2^ We present a rare case of isolated right axillary artery aneurysm in a 13-year-old girl. Parental consent was obtained before reporting this case.

## Case report

A 13-year-old female swimmer with a history of asthma and scoliosis presented to her pediatrician with a several-month history of asymptomatic palpable mass in the right axilla. She reported a viral illness a few months before presentation. The mass was treated initially as reactive lymphadenitis with a course of oral antibiotics. On follow-up, the mass was still present and noted to be pulsatile on examination. The patient reported some mild right-hand tingling with the arms overhead. She denied any history of trauma. She had no family history of aneurysm disease. Duplex ultrasound examination was performed, documenting a 1.5 × 1.5 cm right axillary artery aneurysm without evidence of intraluminal thrombus and with otherwise normal arterial flow throughout the remainder of the upper extremity vessels.

**Fig 1:**
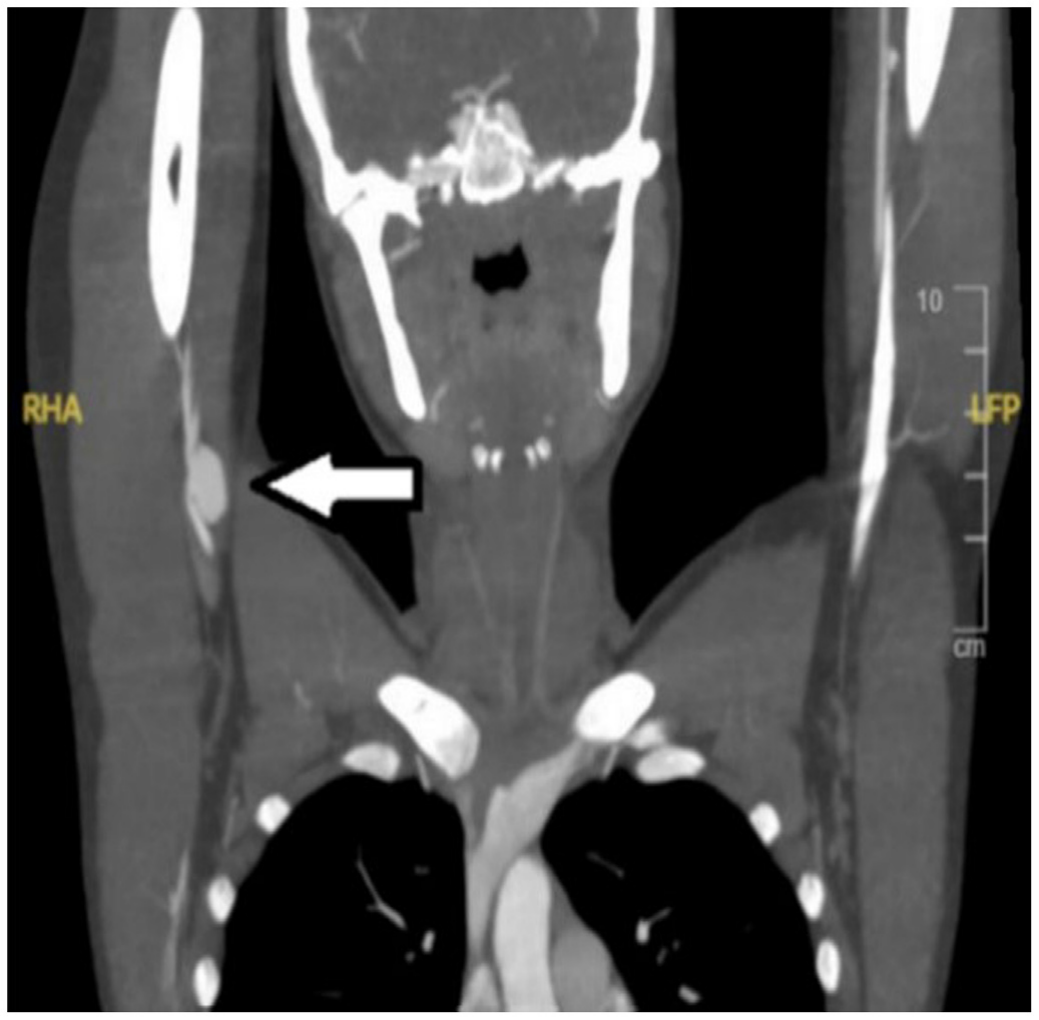
Computed tomography arteriogram demonstrating right axillary artery aneurysm.

An echocardiogram showed no cardiac abnormalities. Magnetic resonance imaging of the chest and abdomen showed no evidence of other aneurysms. Duplex ultrasound examination was repeated with a protocol to evaluate for thoracic outlet compression. This examination was normal, except for the absence of right index finger signal with the arm raised at 180°. A computed tomography arteriogram was performed with arms overhead and did not identify any evidence of arterial compression at the thoracic outlet or by the pectoralis minor muscle. Axillary artery aneurysm anatomy was clearly delineated on computed tomography arteriogram (Fig 1). Genetic testing was performed and showed no evidence of genetic mutation known to be associated with connective tissue disease, but did show a mutation of unknown significance in *LTBP3*. The patient was started on aspirin 40.5 mg/day. Management options for the axillary artery aneurysm were discussed, including conservative therapy, endovascular repair, and open surgical repair. The patient and her parents decided to proceed with operative repair and were advised that although primary repair would be considered intraoperatively, great saphenous vein interposition would be performed if primary repair was not feasible. Preoperative ultrasound mapping of the lower extremities identified a suitable caliber great saphenous vein for native conduit use.

**Fig 2:**
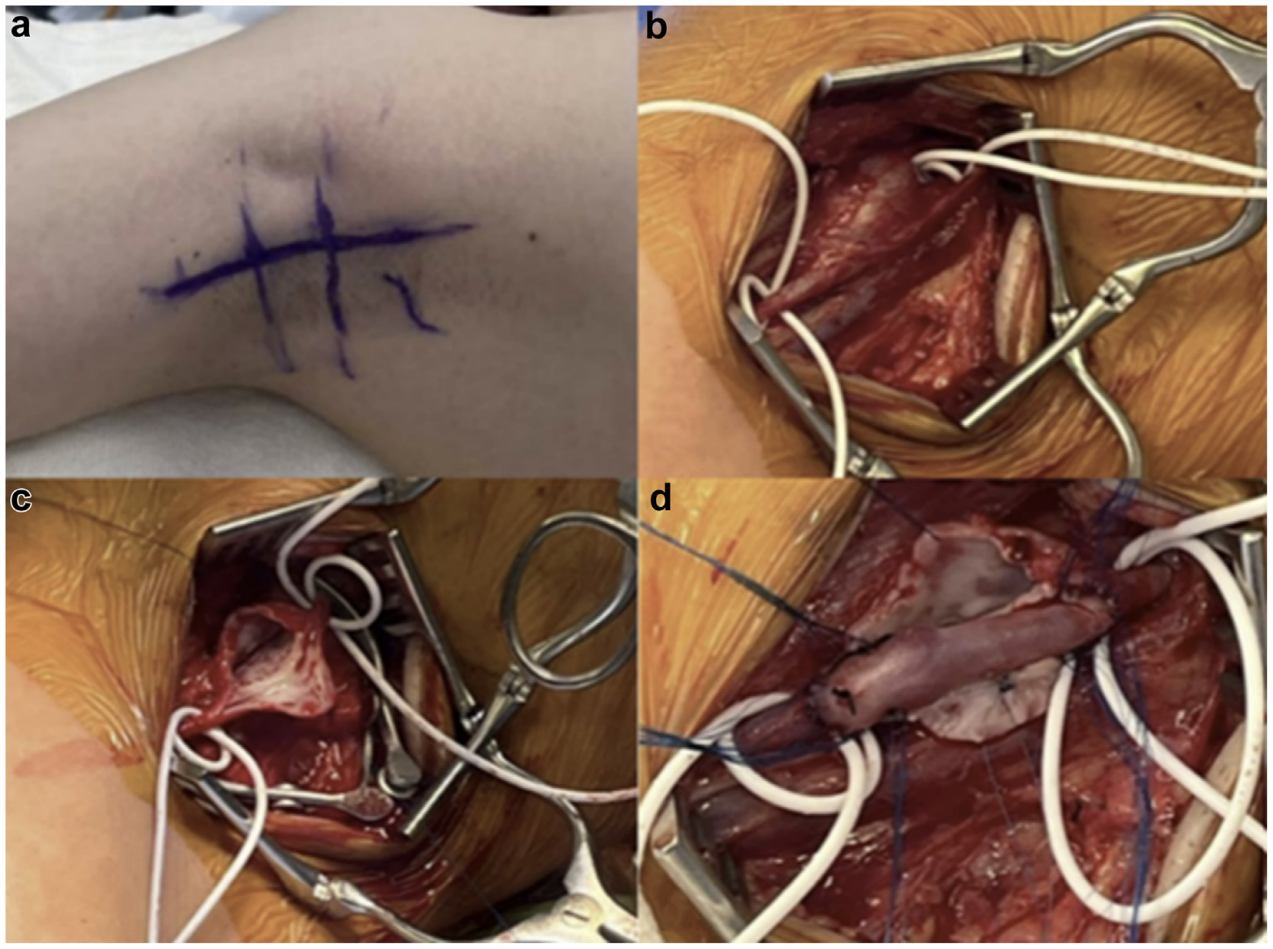
**(A)** Right axilla. **(B)** Axillary artery aneurysm. **(C)** Axillary artery aneurysm after arteriotomy. **(D)** Axillary artery aneurysm after repair with great saphenous vein interposition.

General endotracheal anesthesia was used. The right axilla and the left groin and thigh were prepped and draped into the operative field. Ultrasound imaging was used to map and mark on the patient's skin the location of the axillary artery aneurysm. A curvilinear incision was performed in the axilla and the distal axillary artery and proximal brachial artery were dissected free and encircled with vessel loops proximal and distal to the aneurysmal segment. No evidence of compression of the artery was noted during the dissection. The patient received a bolus of 80 U/kg of intravenous heparin, maintaining the activated clotting time of >200 seconds throughout the case. The artery was clamped proximal and distal to the aneurysm. The aneurysm sac was incised, extending the arteriotomy to healthy appearing artery proximal and distal to the aneurysm. An approximately 4-cm segment of artery required interposition. Primary end-to-end anastomosis was considered, but was felt to be at risk of too much tension; therefore, a segment of left great saphenous vein was harvested from the groin. Arterial interposition was performed with reversed left great saphenous vein using end-end interrupted 6-0 Prolene anastomoses (Fig 2). On completion, a palpable 2+ radial pulse with triphasic doppler signals was noted. The patient recovered uneventfully and was discharged home on postoperative day 1 on aspirin 40.5 mg/day. At the 2-week follow-up, she denied pain or numbness and had palpable radial and ulnar pulses. At the 1- and 9-month follow-up appointments, duplex ultrasound examination of the right upper extremity demonstrated a patent interposition graft and normal arterial flow throughout the upper extremity. Aspirin was discontinued 9 months postoperatively. A surveillance plan for annual ultrasound imaging and clinic evaluation was established. The patient resumed all physical activities including swimming with no limitations.

## Discussion

We report a rare case of isolated right axillary aneurysm in a 13-year-old female patient. Multiple etiologies for pediatric axillary artery aneurysms exist, including trauma, genetic disorders, and systemic diseases.^3^ One retrospective analysis reported on 61 aneurysms in 41 pediatric patients. Using the Sakar classification system, the most common cause of nonaortic arterial aneurysm in this study was trauma (n = 14) and arterial dysplasia (n = 13), with the most common site affected being the renal arteries (n = 26).^4^ In a review of literature, only 14 cases of pediatric axillary aneurysms were identified.^2^

The workup of aneurysms in the pediatric population is essential in management. True aneurysms in the pediatric patient are organized using the using a classification system proposed by Sarkar et al.^1^ Categories include (I) arterial infection, (II) giant cell aortoarteritis, (III) autoimmune connective tissue disease, (IV) Kawasaki disease, (V) Ehlers-Danlos syndrome or Marfan's syndrome, (VI) other forms of noninflammatory medial degeneration, (VII) arterial dysplasia, (VIII) congenital-idiopathic factors, and (IX) false aneurysms associated with extravascular events causing vessel wall injury or disruption.^1^ Using this classification system can help to guide the initial workup in a pediatric patient with a newly diagnosed aneurysm. In our case, the patient had a prior history of scoliosis and reported hypermobility in her joints which raised suspicion for connective tissue disease. Genetic testing was performed to rule out connective tissue disorder as well as any autoimmune disease.

The management of axillary aneurysms is guided by published case reports, most suggesting surgical repair using venous graft interposition.^4^, ^5^, ^6^, ^7^ Caution should be taken in operative technique to avoid tension on the anastomosis and allow for vessel growth in the child.^2^ In this case, we elected for a surgical approach with great saphenous vein interposition. The great saphenous vein is the conduit of choice when interposition is performed. The use of prosthetic grafts in arterial reconstruction for aneurysmal disease in children has been reported but, given suboptimal long-term patency and potential mismatch as the child grows, it is not typically the first-line treatment choice.^7^, ^8^, ^9^ One report documented long-term graft patency of 100% at 3.20 ± 0.41 years, however long term-term graft patency data has not been well-established in the pediatric population.^10^^,^^11^

Established guidelines for optimal management after aneurysm repair in pediatric patients is limited. There is some evidence reported on the use of aspirin as antiplatelet therapy in children, as the risk of arterial thrombosis may exceed that of complications of aspirin therapy such as Reyes syndrome. It has been shown that preoperative aspirin therapy continuing for 6 months postoperatively decreases the risk of thrombus formation in small caliber arteries.^4^^,^^9^ In addition to antiplatelet therapy, routine duplex ultrasound surveillance should be performed to assess graft patency at regular intervals, like surveillance protocols for arterial bypass in other settings.

## Conclusions

Nonaortic aneurysms, especially axillary aneurysms, are rare in children and a thorough workup is needed to determine the etiology. Management is currently guided by case reports with most support for surgical repair with venous interposition although primary end-to-end anastomosis has been reported. Careful follow-up as the child grows is needed to ensure the integrity of the repair.

## Funding

None.

## Disclosures

None.
